# “We are the ones who will have to make the change”: Cuban health cooperation and the integration of Cuban medical graduates into practice in the Pacific

**DOI:** 10.1186/s12960-023-00822-8

**Published:** 2023-05-03

**Authors:** Sharon McLennan, Cristine Werle

**Affiliations:** School of People, Environment and Planning, Massey University Manawatu, Private Bag 11 222, Palmerston North, 4442 New Zealand

**Keywords:** Cuban cooperation, Pacific health workforce, Medical Internship, Solomon Islands, Kiribati

## Abstract

**Background:**

This paper responds to Asante et al. (in Hum Resour Health, 2014), providing an updated picture of the impacts of Cuban medical training in the Pacific region based on research carried out in 2019–2021, which focused on the experiences of Pacific Island doctors trained in Cuba and their integration into practice in their home countries.

**Methods:**

The research focussed on two case studies—Solomon Islands and Kiribati. Study methods for this research included multi-sited ethnographic methods and semi-structured interviews as well as qualitative analysis of policy documents, reports, and media sources.

**Results:**

The Cuban health assistance programme has had a significant impact on the medical workforce in the Pacific region increasing the number of doctors employed by Pacific Ministries of Health between 2012 and 2019. Qualitatively, there have been some notable improvements in the medical workforce and health delivery over this period. However, the integration of the Cuban-trained doctors into practise has been challenging, with criticisms of their clinical, procedural and communication skills, and the need for the rapid development of bridging and internship training programmes (ITPs) which were inadequately planned for at the outset of the programme.

**Conclusions:**

The Cuban programme in the Pacific is an important model of development assistance for health in the region. While Cuba’s offer of scholarships was the trigger for a range of positive outcomes, the success of the programme has relied on input from a range of actors including support from other governments and institutions, and much hard work by the graduates themselves, often in the face of considerable criticism. Key impacts of the programme to date include the raw increase in the number of doctors and the development of the ITPs and career pathways for the graduates, although this has also led to the reorientation of Cuban graduates from preventative to curative health. There is considerable potential for these graduates to contribute to improved health outcomes across the region, particularly if their primary and preventative health care skills are utilised.

## Background

Over the past decade there has been a quiet revolution in health care in the Pacific region as over 200 newly graduated doctors arrived home from La Escuela Latinoamericana de Medicina (ELAM)—the Latin American School of Medicine in Cuba, doubling the number of doctors in some Pacific nations. Trained in a medical programme that emphasises primary and public health care, these doctors offer much needed skills in a region that faces serious challenges including the unfinished communicable disease (CD) agenda; a rapidly rising non-communicable disease (NCD) epidemic; and the impacts of climate change [31], burdens compounded by the COVID-19 pandemic. One of the most pressing needs is for human resources for health (HRH). The Cuban health assistance programme was a key intervention to address this need. Over the course of the programme Cuba sent health personnel to Pacific Island countries (PICs), and, more significantly, provided scholarships for training over 200 medical students from nine PICs.

### Health challenges in the Pacific

Many PICs have made significant health gains in recent years, including improvements in vaccine-preventable diseases rates, reductions in maternal and child mortality rates, and improvements in the control of NCDs [31]. Primary health care (PHC) and universal health coverage (UHC) have been a particular focus of the Healthy Islands vision [6, 32]. However, the World Health Organization (WHO) 2018–2022 Cooperation Strategy for the Pacific notes that PICs faces considerable challenges including CDs, the NCD epidemic; and the health impacts of climate change [31], burdens that are likely to be compounded by the COVID-19 pandemic. In addition, most Pacific Island countries face considerable challenges in delivering comprehensive health services, including environmental vulnerability, the difficulties inherent in the small size and remoteness of PICs, including limited resources, and increasing poverty and inequality [8, 23, 24, 31].

A major contributor to poor health in PICs is the unevenness of health services. PHC forms the backbone of the service delivery system in many PICs; however, small and isolated populations, workforce limitations, weak governance, inadequate health facilities, and supply chain and logistics difficulties challenge PHC delivery [6]. PICs face challenges in recruiting and retaining health personnel, although there is considerable variation in the density of skilled health workers across the region.

#### Human resources for health and medical training in the Pacific

A key issue in capacity building for health and health resilience is human resources. A sustainable medical workforce is one of the foundational building blocks of health systems and a prerequisite for health system resilience [3]*.* However, the planning and management of human resources for health (HRH) remains the least developed aspect of health systems policy and development in many low and middle income countries [34]*.* This is particularly the case for PICs, with several failing to meet the WHO minimum recommendation of 2.3 health workers per 1000 population.

As an important component of the health workforce, key priority area for PICs has been to increase the number of doctors [22]. Traditionally, undergraduate medical education in PICs was provided by Fiji National University (FNU), the University of Papua New Guinea, and (limited) places in Australian and New Zealand universities [22]. Historically medical graduates predominantly undertook medical internships in Fiji with smaller internship programmes in Palau, Solomon Islands, Vanuatu, Samoa and Tonga [12, 13]. However, medical education and postgraduate training systems in the region lack the capacity to produce sufficient numbers of clinicians ([15] p. 94; [20]). In addition, the distribution of the workforce is unequal with the majority of the region’s doctors concentrated in the central referral hospitals.

### Cuban cooperation for health

Cooperation with Cuba has emerged as a key means to address this medical workforce crisis. Cooperation in health has been an essential component of Cuba’s foreign policy for more than five decades [26]. While Cuba’s medical cooperation is most visible during crisis events, it is usually directed towards long-term public health, and community-health. This focus on primary-care and public health is particularly significant. Cuba has a reputation for having a relatively well performing health system that provides Cubans with better access to healthcare and longer life expectancies than many developed countries ([15], p. 94).

Up until 1999 Cuban medical cooperation was largely focussed on sending Cuban health professionals to serve in other nations. Following Hurricanes Georges and Mitch in 1998. Cuban medical professionals were sent to work in Haiti and Central America, but the government soon realised it was not sustainable for Cuban doctors to staff foreign health systems indefinitely and that “the ideal provider is a well-trained, home-grown health professional” ([10], p. 86). To address this Cuba created medical scholarships for international students. Recipients study at ELAM for free under the agreement that upon completion of their studies they return home to serve their communities [10, 11].

Cuba’s commitment to Universal Health Care (UHC) and recognition of the value of PHC is deeply embedded in the education provided by ELAM [10, 11, 16]. From the beginning, ELAM’s goals reflected Cuba’s commitment to solidarity, internationalism, and sustainable development, with programmes that aimed to build health care capacity in countries around the globe, educating doctors primarily for public service who have competencies in comprehensive primary care [18, 28]. ELAM is 6-year medical school “providing hands-on learning about social determinants’ effects on health; sharing responsibility for addressing spatial inequalities and delivering equitable, accessible services; going beyond pedagogical innovations to include integration of graduates into local health systems” ([10], p. 86). While Cuban brigades continue to offer support, the scholarship program is viewed as more empowering to local communities, and better equipped to serve their long-term needs.

#### Cuban cooperation in the Pacific

The first nation in the wider Pacific region to receive Cuban assistance was the newly independent nation of Timor Leste. Over a decade Cuba trained nearly 1000 doctors at ELAM and deployed 300 Cuban doctors to provide medical care and to supervise and further train students and new doctors. This programme spread across the South Pacific, with Cuban doctors serving in Kiribati, Nauru, Solomon Islands, Tuvalu and Vanuatu between 1999 and 2017 [7]. In addition nearly 250 medical students from the Cook Islands (1), Fiji (32), Kiribati (33), Nauru (4), Palau (13), Solomon Islands (104), Tonga (14), Tuvalu (22) and Vanuatu (21) were given scholarships to study in Cuba [1]. This research is the first to specifically explore Cuban cooperation in Pacific island nations; however, the impact of the return of the graduates is seen in studies of medical internships [21], medical and postgraduate training in the region [15, 22], and of the UHC-related priorities of Pacific health authorities [6]. These studies all note both the impact on the health workforce and the challenges of integrating the graduates into practice.

## Research aims and objectives

The Cuban health assistance programme in the Pacific was the focus of a 2014 article by Asante et al. which found that despite the small scale of Cuban engagement with PICs, the programme presented several opportunities for health systems strengthening in the region, allowing PICs to increase their health workforce numbers at relatively low cost, but noted that the rapid growth in the health workforce would be challenging. However, there has been little follow-up on the impacts of this on HRH in the region.

This paper, therefore, responds to Asante et al. [2], providing an updated picture of the impacts of Cuban medical training in the Pacific region based on research carried out in 2019–2021. The aim of this research was to explore how the Cuban approach to medical cooperation contributes to health and development in the Pacific**.** The specific objectives were to:

1. Explore the Cuban approach to health, particularly in the Pacific.
2. Investigate the scope of Cuban medical assistance in the Pacific.
3. Identify the impacts of Cuban medical assistance in the Pacific

## Methods

To obtain an in-depth picture of the contribution of the Cuban programme, this qualitative study focussed on two case studies—Solomon Islands and Kiribati. These two cases were chosen as having the highest proportion of Cuban medical scholarships given in relation to the number of doctors practicing in the country at the time the programme began. Both these countries were provided with more Cuban medical scholarships than there were doctors in country at the commencement of the programme. In 2005, Solomon Islands had 89 doctors, and between 2007 and 2019 sent 104 students to Cuba. Kiribati had 25 doctors in 2005 and sent 33 students to Cuba between 2007 and 2019 [1, 35]. Vanuatu also had a significant number of scholarships, although less than the selected cases, and re-developed their Internship Training Programmes (ITP) in response to the returning graduates; however, the inclusion of another case was beyond the scope of this study.

Kiribati and Solomon Islands also each developed (Kiribati) and re-developed (Solomon Islands) ITPs in response to the return of those graduates. This provided insights into the impacts of the medical scholarships, the experiences of Pacific Island doctors trained in Cuba, and their integration into practice.

### Sampling and data collection

Study methods for this research included multi-sited ethnographic methods and interviews as well as qualitative analysis of policy documents, reports, and media sources. Fieldwork in Kiribati in 2019–2020 included non-participant observation at Tungaru Central Hospital (TCH), and semi-structured interviews and focus groups (see Table 1). Twenty-two in-depth interviews were conducted with Cuban and Cuban-trained doctors, Kiribati Internship Training Programme (KITP) supervisors and advisors, nursing representatives and health workers and members of the community [29, 30]. A further eight Cuban-trained doctors participated in a focus group. A focus group was not originally planned but was proposed during the research as time was a constraint due to the heavy work schedules of participants. Three follow-up online interviews with Cuban-trained doctors who had completed the KITP were conducted in 2021.

**Table 1:** Participants

Participants	Country association	Interviews	Follow-up interviews	Focus Group
Cuban-trained interns/registrars	Kiribati	6	3*	8
Cuban doctors	Kiribati	2	–	–
Supervisors and medical trainers	Kiribati Solomon Is	5 + 2* 6*	–	–
ITP programme coordinators	Solomon Is	4*	–	–
Volunteer coordinator	Australia/Solomon Is	1*		
Advisors and consultants	All	2*		
Nursing representatives and other health workers	Kiribati	4	–	–
Community members	Kiribati	3	–	–

^*^Interviewed via Zoom. The remainder were face-to-face interviews

Cuban and Cuban-trained doctors were central sources of information for this research; however, we also collected data from others who worked with these doctors. Sampling for this project was purposive and opportunistic using snowball methods. This was a culturally appropriate and a feasible means of recruitment in a small place like Tarawa [14, 27], where the people who are or have been engaged with the Cuban programme are small in number and knew each other. The focus group session was attended by the cohort of Cuban-trained doctors undertaking the KITP at the time of the research, a group composed of interns from Kiribati (3), Tuvalu (3) and Nauru (2). Participation throughout the process was open to any Cuban-trained doctor, or other health professional or community member with an interest in the programme so there were no exclusion criteria.

The non-participant observation at Tungaru hospital was carried out by one of the researchers with a local research assistant who, with permission from the hospital and relevant staff, spent a month at the hospital, sitting in a corner of the ward and observing, or following individual staff around, observing hospital routines, roles, and responsibilities. This provided important insights into the work of the Cuban-trained interns and doctors as well as assisting with building relationships for research recruitment.

Unfortunately, similar fieldwork in Solomon Islands could not proceed due to the pandemic as the researchers were unable to complete planned fieldwork or interview Solomon Island health professionals, but data were gathered from reports and other grey literature, and online interviews with 13 Australian volunteer medical trainers and experts associated with the Solomon Islands Internship Training Programme (SIITP). Recruitment for these interviews was also via purposive recruitment (experts and advisors) and snowballing (SIITP supervisors and volunteers), with participants identified via word of mouth. The criteria for inclusion were based on participant’s involvement with the SIITP, with all participants having spent time working in the internship programme or as key advisors.

Other data collection methods contributing to the study included analysis of over 100 policy documents, reports, and media sources (see Table 2). Initially these sources were identified through an online search for the terms “Cuba”, “Kiribati” OR “Solomon Islands”, “health” and “doctors”. Data sourced this way included national health strategies and policies, publicly available reports, and media publications. Over the course of the research, we also obtained unpublished donor reports, meeting summaries and programme reviews from participants.

**Table 2:** Documents analysed

Document type	Number
National health strategies	2
Government policy and reports	7
Regional health and development strategies	5
ITP reports and reviews	6
Government response to ITP review	1
Other programme review	2
Pacific Heads of Health meeting report	2
MEDICC (Medical Education Cooperation with Cuba) reports	4
MINREX (Ministerio de Relaciones Exteriores, Cuba) publications	5
Pacific media reports	38
Cuban media reports	6
Other media reports	45
Bilateral and multilateral donors’ reports	9
Working papers and meeting summaries	5
World Health Organization reports	11
Presentation	1

### Data collection instruments

Interviews were key instruments of data collection and provided meaningful verbal and non-verbal qualitative data. This method was considered appropriate as Kiribati has a strong oral culture, because we were interested in eliciting specific descriptions, feelings, thoughts and opinions about health care and the transition of graduates returning from Cuba into medical practice in the Pacific. The interview schedule for Cuban-trained doctors (including guidelines for the focus group) included key questions on perceptions of Cuban training, transition to practice and the ITP, and future plans. Interviews for supervisors and advisors covered their role, experiences with Cuban trained doctors, prior knowledge and the programme and learning since, and recommendations, although these were adjusted for each interview depending on the role of the interviewee.

### Data analysis

This study used thematic content analysis for both interview and documentary sources. Initial data analysis was undertaken by the postgraduate student with support from the primary researcher. During this process a basic coding framework was developed based on the research objectives and documentary sources. The recorded interviews and focus group from the Kiribati case study were transcribed verbatim, and these were coded to the framework, supplemented with inductive, open coding. The primary researcher then analysed the Solomon Islands data in a similar manner, reviewing and re-analysinganalysing the earlier data using constant comparative methods to refine the codes and to develop key themes that cut across both case studies.

## Results

### The impacts of Cuban cooperation in the Pacific

It is now a decade, since the first Cuban-trained graduates returned home. While there are no official reports on the impact of the Cuban programme, many key health statistics have continued to improve across the region. These improvements cannot be conclusively linked to the Cuban programme, particularly as they continue a trajectory of improvements predating 2006 and the number of doctors is just one component. However, it is clear from this research the Cuban programme is having an impact on the medical workforce and health care delivery.

**Fig. 1:**
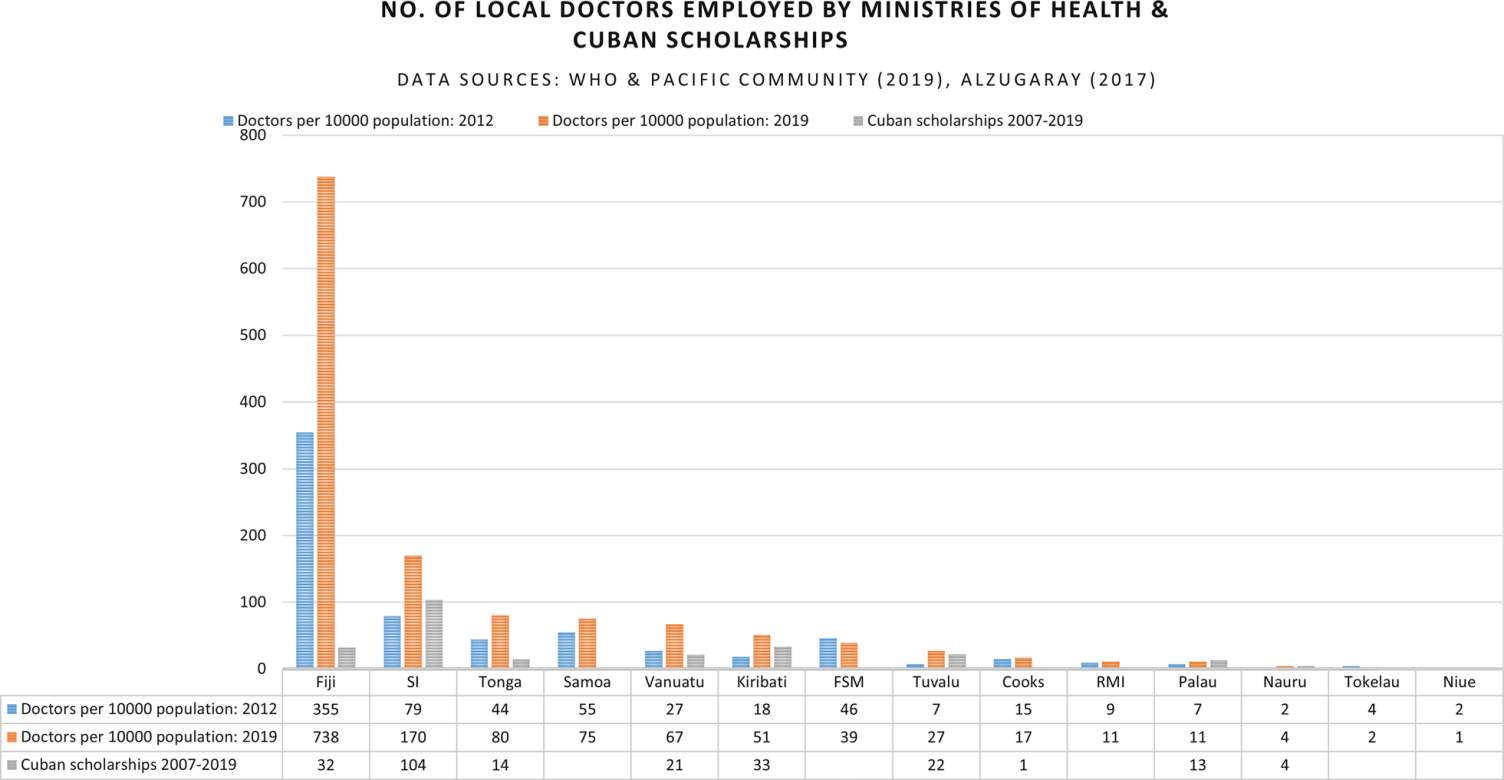
No. of local doctors employed by ministries of health and Cuban scholarships

Figure 1, drawn from data presented at the thirteenth Pacific Health Ministers meeting in 2019, demonstrates this impact, showing a significant increase in the number of doctors employed by several Pacific Ministries of Health between 2012 and 2019 [32], alongside the number of Cuban scholarships given to Pacific students. Although the figures for doctors employed is per 10,000 population (and hence not directly comparable with the number of scholarships), the figure shows the impact of Cuban scholarships on the number of doctors in PICs.

Qualitatively, there have been some notable improvements in health delivery with the return of the Cuban-trained doctors. Early in the programme, Cuban-trained doctors were identified as a driver in the strengthening of Kiribati’s health system [17], enabling doctors to spend more time with patients and deliver higher value care. This improvement was also noted by research participants at TCH who noted that since the beginning of the Cuban programme the number of departments at TCH that had a full team (nurse, medical intern, registrar, specialist) had increased and the ability of the hospital to respond more promptly to afterhours medical care and emergency situations had improved:

In the past, before the start of KITP, if you are lucky you get seen by the doctor once a week. Now patients get seen by the doctors 2 or 3 times a day, so patients are commenting about that. (KITP Supervisor, interview)

While some changes are evident, the Cuban trained doctors are still early in their careers, and the true impact may be well into the future. It is also not yet possible to ascertain what contribution these doctors are making to the COVID-19 pandemic response. However, the apparent success of the Cuban programme has been hard fought, with the graduates facing a range of challenges and critiques along their journey.

### Concerns about Cuban-trained graduates

A key concern in the early days of the Cuban programme was the integration of Cuban-trained graduates into the health systems of PICs. These were not unfounded. By the time the medical graduates arrive home they are already 7 years into their journey to become doctors. They began that journey by travelling over 10000 km to Cuba, then spent a year learning Spanish, completing the premedical program, and acclimating to Cuban culture before starting 6 years of medical training. On their return home the graduates faced the challenges of completing their internship, a process complicated by language, criticism of clinical skills and insufficient understanding of Cuba and Cuban medical training within Pacific health systems.

Although the graduates had a good basic medical knowledge, senior medical staff noted that many could not perform basic procedures. Many did poorly on initial exams and were found to lack the clinical, procedural and communication skills necessary for safe and effective medical practice:

We had around 30 Cuban graduates coming back that first year. And none of them passed [the clinical exam]… we said that they were probably like a year two standard from FNU. Some of the theory was OK, but they had no practical skills (Medical Education Consultant, Solomon Islands, interview)

This reflects the findings of Sweet et al (2018), p. 3, in a project report on the design and delivery of professional education workshops in the Pacific analysed as part of the data set for this research, who noted that many Cuban-trained interns had difficulty inserting intravenous lines, writing detailed prescriptions, or using medical English.

Some of the harshest critiques of the Cuban-trained graduates came from established members of the medical community:

Before they [the Cuban graduates] came back, there was already this decision made that… they would be hopeless and poorly trained... And then there’s just been this constant scrutiny in the media, just this terrible kind of negative attitude and it came from…within the medical institutions… and that ‘old guard’… they’ve had to deal with some terrible prejudice (Medical Consultant, SIITP, interview)

The prejudice and criticism of the medical establishment, politicians and media had flow on impacts for patients trust:

When the first cohort came back and went into the bridging program, there was a lot of chatter around the community that they were hopeless. They were no good, that the training was inadequate... And I heard anecdotal stories of people saying, don't touch me, you're a Cuban. I won’t have you treat me, those sorts of things. (Medical trainer 1, SIITP, interview)

This distrust was evident in a letter to the editor of the *Solomon Star* in September 2020 which questioned the capabilities of Cuban-trained doctors and referred to them as “bush doctors”. There were several reasons underlying these concerns. First, there are some fundamental differences between the Cuban health system and the context graduates need to practice in on their return to the Pacific. This includes basic differences in training. Cuban medical training is highly focussed on preventative and primary health care, in contrast to the Western-oriented systems that Pacific doctors have traditionally trained in, which tend to have a curative focus. As the quote below indicates, this means that Cuban-trained graduates may have superior community and PHC primary health training but may lack knowledge and skills in secondary and tertiary care.

They (students at ELAM) spend a couple years at the main school in Havana and often they spend the next three, four years at a provincial hospital… they just aren’t getting the tertiary and specialist experience that you get in most medical education. At the same time … the strength of Cuba’s approach is in preventative care and community care. So they’re very, very good at going into community and doing community health assessments and working from the ground up… this is the problem, that in part, their education lacks some clinical experience and skills, but more so it also is a problem of the structure of health services in the Pacific (Medical trainer 2, SIITP, interview).

The gap between Cuban training and the Pacific health systems in which the graduates need to work is exacerbated by differences in HRH and role delineation. While the Cuban health system may be considered low resource in terms of cost, it is highly resourced in HRH. This means roles within the Cuban system are more prescribed and medical and allied health teams much larger. Therefore, many routine tasks such as cannulation are done by nurses and other health workers in Cuba, and a medical student may never get the opportunity to practice. In the Pacific, HRH resources and low and teams small, so graduates need to have a broad range of clinical skills.

In addition, at home in the Pacific graduates are routinely exposed to—and need to be able to respond to—health conditions not regularly seen in Cuba:

In Cuba you wouldn't see a case of malaria during your undergraduate training and whereas on your first day as an intern working in the emergency department at NRH (National Referral Hospital) in Honiara the first patient you would see would be somebody with severe and complicated malaria. In Kiribati or Tuvalu, the first patient you see on your first day working in outpatients with a cough, high chance they'd have TB, rather than just somebody with a sniffle. (Consultant & Advisor to KITP & SIITP, interview)

Differences in context and training were exacerbated by language difficulties. Medical training in Cuba occurs in Spanish so Cuban-trained interns also struggled with medical English. Many believed that their lack of ability to use medical English influenced perceptions about their medical competence:

When we started our internship, we hardly communicated in English. It’s hard for us to communicate directly in English, especially solving problems and things like that… We know things but the way we express it and translate it, our supervisors don’t understand what we are trying to say. (Cuban-trained intern, Kiribati, interview)

To address concerns about language and clinical skills, and to prepare medical graduates who had trained outside of Fiji and PNG for the ITPs, bridging courses were established in Kiribati and Solomon Islands. Kiribati has a 3-month pre-internship bridging course, while Solomon Islands bridging course is a full year, but each aims to help medical graduates consolidate, expand and apply their knowledge and skills in their home context [19, 25].

### Evolution of the ITPs

The development of ITPs was not an original focus for the research, but it became clear that the return of such significant numbers of graduates from Cuba presented an urgent challenge for Health Ministries, and the establishment of these programmes was key to the integration of Cuban graduates into practice. The following brief history of the ITPs is drawn from interviews with key advisors to the Kiribati and Solomon Islands health ministries, and ITP planning and review documents and reports analysed in the documentary data set for this research (including [4, 5, 9, 12, 13, 22, 25]).

Prior to 2014, medical graduates from across the Pacific completed internships in Fiji. However, around the same time that the first cohort of Cuban-trained doctors were due to return, Fiji closed its internship programmes to foreign-trained medical graduates (FTMGs). This created a major problem for smaller Pacific states with hundreds of Pacific students enrolled in medical training outside Fiji, including ELAM, I-Shou University (Taiwan), Oceania University of Medicine (Samoa) and to a lesser extent institutions from Georgia, Kazakhstan, Russia, and Morocco.

Planning for the return of the Cuban-trained and other FTMGs, therefore, began after the first scholarships had been awarded, as Health Ministries and donors scrambled to develop pathways to practice for these graduates. Planning began with the establishment of the Strengthening Specialised Clinical Services in the Pacific (SSCSiP) programme in 2011. This was funded by the Australian Department of Foreign Affairs and Trade (DFAT) and hosted at FNU. SSCSiP aimed to strengthen country-level HRH planning and capacity [22]. Early work on the ITPs in the region also emerged from the WHO’s Pacific Human Resources for Health Alliance (PHRHA) which had a focus on workforce planning, including medical workforce planning.

The KITP was established in 2013, implemented by the Kiribati Ministry of Health and Medical Services (MHMS), with technical guidance from Fiji, DFAT, and the WHO. It is open to FTMGs from Kiribati, Tuvalu and Nauru and has trained over 60 doctors since 2013 [25]. Graduates commence with the Bridging Program, before completing a minimum of 18 months of supervised clinical, community and public health practice. The KITP was the first Pacific ITP outside Fiji to be formally endorsed by the Postgraduate Committee of the College of Medicine, Nursing and Health Sciences of FNU, so KITP graduates can enter postgraduate study at FNU. An independent external review in 2018 found that the KITP had “a reputation as a well-structured Internship program that produces well-trained and well-balanced medical officers” [25], p. 8).

The SIITP was established in 2014. It includes the 1-year bridging program and a 2-year internship programme with all trainees rotating through specialist and sub-specialty rotations. The programme is administered by the and taught by the Solomon Islands Medical Partnerships for Learning, Education and Research (SIMPLER)—previously known as the Solomon Islands Graduate Internship Supervision and Support Project (SIGISSP), a collaboration between the Australian Volunteers Program (AVI), DFAT, Solomon Islands MHMS and the NRH, Honiara [13]. A 2018 review found a consensus of opinion that the programme is successful in improving the essential clinical skills of medical graduates and their capacity to make independent decisions [9], and SIITP graduates can now also go on to do postgraduate study in Fiji.

Access to postgraduate study for Cuban-trained doctors was a key outcome for the ITPs; however, many research participants noted that the training provided by the ITPs to upskill and re-orient Cuban-trained graduates into secondary and tertiary care and to enable career progression tends to shift Cuban-trained graduates from proactive to reactive health workers. As a consultant to the KITP noted:

The fundamental premise of the Cuban undergraduate training is ‘I go out to serve my community’, and some of them are not specifically philosophically aligned with hospital specialisation. But in the Pacific, there is a shortage, particularly in certain specialties like internal medicine and paediatrics and the door needs to be open for people to have a grounded re-entry experience in their country after medical school outside the region. (Consultant & Advisor to KITP & SIITP, interview)

Unfortunately, this has meant that the Cuban-trained doctor’s knowledge and experience in primary and preventative health is often overlooked and underexplored. Cuban-trained doctors in this research and elsewhere [9], p. 8) often expressed frustration that they did not have time to fully utilise their public health skills. For example, several noted that the Cuban practice of Comprehensive General Medicine is well-aligned with holistic understandings of health in PICs, and the utilisation of the Cuban Continuous Assessment and Risk Evaluation (CARE) tool could markedly improve health data and enrich epidemiological analysis. It could also contribute to the design of more responsive health policies and of targeted public health initiatives. Maternal, new-born, and child health is another area that graduates identified could potentially be improved.

## Discussion

In 2014 Asante et al. concluded that Cuban engagement with PICs presented several opportunities for health systems strengthening, allowing PICs to increase their health workforce numbers at relatively low cost and to extend delivery of health services to remote areas. This research has shown that the Cuban programme has significantly increased the medical health workforce and improved quality of care in many PICs, although the spread of services into remote areas has been slow due to the need to upskill Cuban-trained doctors for work in secondary and tertiary care and to allow graduates to be able to specialise. Work is currently underway to develop to develop qualifications and pathways to practice in family, community and rural hospital medicine (for example [15], however, the impact Cuban-trained graduates will have on rural and remote health services is yet to be seen, and should be a focus for future research.

Asante et al. [2] also noted key challenges with the increase in the number of medical doctors including concerns about the integration of Cuban-trained medical graduates, and the implications for training providers and donor assistance. In terms of the integration of Cuban-trained graduates, challenges related to skills and competency have been largely resolved through the rapid and successful development of bridging and internship programmes, and most graduates are now working as doctors in their respective home countries. However, this research reflects ongoing questions about health workforce planning in the Pacific region. Scholarships were awarded and students sent to Cuba without adequate planning for their return, internship, and integration into practice, with medical training essentially disconnected from national workforce planning (see also Condon, Duvivier, Kirition, Kafoa, Mckimm, & Roberts, 2013, p. 2). While this concern was flagged by Asante et al. [2] and efforts to address this were evident in the early 2010s through the PHRHA and Health Ministries in the region, the development of the ITPs and other initiatives for career progression were largely reactive. Moreover, the challenge of completing the graduates’ medical internships required considerable support from donors and other training providers including FNU, DFAT and New Zealand’s Ministry of Foreign Affairs and Trade (MFAT).

This support means that the pathway from ELAM, through bridging and internship to practise and on to postgraduate study and specialisation is clear. However, the constraints of current health structures, and the need to back-fill medical positions in the secondary and tertiary sector along with the responsibility to ensure career options and specialisation pathways have colluded to shape the graduates into reactive rather than proactive health workers. Despite this, the desire to improve public and community health remains within the graduates. As the Cuban and ITP programmes mature it is imperative that the knowledge and skills gained by Cuban graduates at all steps of their journey is utilised to bring better health outcomes for the people of the Pacific.

### Limitations

The researchers note the lack of Solomon Island participants in this study as the result of pandemic restrictions is a limitation, particularly in discussions of intern experiences. This will be addressed in planned future research with the SIITP.

## Conclusion

During the hard days when we were seeing people not meeting their competency criteria during their quarterly assessments and having to extend or even repeat their rotation we thought, Oh, my goodness. But then, in retrospect, having seen [graduates] now running the emergency department, returning from Fiji with postgrad qualifications and so on is clear that you know in purely backfilling terms… Cuba's done the Pacific a favour. (Consultant & Advisor to KITP & SIITP, interview)

They've got this amazing new workforce that that still retain that strong idea of service that they were trained in Cuba, that strong sense of saving a community and solidarity and all of those things which…. perhaps the old guard tried to beat out of them... what terrific assets for the country… (Consultant & Advisor to SIITP, interview)

These quotes from two Australian advisors to the ITPs summarise both the benefits and challenges of the Cuban programme. In traversing the geographical distance between Cuba and the Pacific and navigating the assimilation into practice at home, these graduates also traversed a border between very different health systems and have found themselves in systems that remain very centralised, and where there has been longstanding shortages and a high need for doctors with clinical and curative focus. The ability of Ministries of Health to address this and to utilise the primary and preventative health expertise of Cuban-trained doctors resource remains an open question, but the final word on this goes to one of the graduates, who noted that:

We are the pioneers, and we are the ones who will have to make the change. We are here and we are still growing, maybe in 10 years’ time you can come back and see what happens. I’m pretty sure we each have in our minds how to improve and to change, and to make health in general better. (Cuban-trained medical graduate, interview)

## Data Availability

Anonymised data for this research can be obtained from the researcher on request.

## Abbreviations

AVI: Australian Volunteers International
CD: Communicable Disease
DFAT: Department of Foreign Affairs and Trade (Australia)
ELAM: Escuela Latinoamericana de Medicina (Latin American School of Medicine)
FNU: Fiji National University
FTMG: Foreign-trained medical graduate
HRH: Human resources for health
ITP: Internship Training Programme
KITP: Kiribati Internship Training Programme
MFAT: Ministry of Foreign Affairs and Trade (New Zealand)
MHMS: Ministry of Health and Medical Services (Kiribati, Solomon Islands)
NCD: Non-Communicable Disease
NRH: National Referral Hospital (Solomon Islands)
PHC: Primary Health Care
PHRHA: Pacific Human Resources for Health Alliance
PICs: Pacific Island Countries
SIITP: Solomon Islands Internship Training Programme
SIMPLER: Solomon Islands Medical Partnerships for Learning, Education and Research
SIGISSP: Solomon Islands Graduate Internship Supervision and Support Project (Predecessor to SIMPLER)
SSCSiP: Strengthening Specialised Clinical Services in the Pacific
TCH: Tungaru Central Hospital (Kiribati)
UHC: Universal Health Care
WHO: World Health Organization
